# Unravelling the Actin Cytoskeleton: A New Competitive Edge?

**DOI:** 10.1016/j.tcb.2016.04.001

**Published:** 2016-08

**Authors:** Andrew J. Davidson, Will Wood

**Affiliations:** 1 School of Cellular and Molecular Medicine, Faculty of Biomedical Sciences, Biomedical Science Building, University of Bristol, University Walk, Bristol, BS8 1TD, UK

**Keywords:** actin, cytoskeleton, competition, chemotaxis, lamellipod, filopod

## Abstract

Dynamic rearrangements in the actin cytoskeleton underlie a wide range of cell behaviours, which in turn contribute to many aspects of human health including embryogenesis, cancer metastasis, wound healing, and inflammation. Precise control of the actin cytoskeleton requires the coordinated activity of a diverse set of different actin regulators. However, our current understanding of the actin cytoskeleton has focused on how individual actin regulatory pathways function in isolation from one another. Recently, competition has emerged as a means by which different actin assembly factors can influence each other's activity at the cellular level. Here such findings will be used to explore the possibility that competition within the actin cytoskeleton confers cellular plasticity and the ability to prioritise multiple conflicting stimuli.

## Trends

Cells maintain a dynamic actin cytoskeleton by carefully balancing the activities of a diverse collection of actin regulators. Recent findings suggest that key actin assembly factors limit one another through competition over a finite pool of G-actin.

Increasing or decreasing cellular G-actin influences the type of F-actin network generated. The actin monomer binding protein profilin is responsible for proportioning how much G-actin is available to each assembly factor.

Cytoskeletal competition appears universally conserved from yeast to human.

Competition ensures cytoskeletal homeostasis and integration/coordination between the different actin regulatory pathways to support dynamic cell behaviour.

## Introduction to the Competition

Cells interact with one another and their environment through precise control of their actin cytoskeleton. This is achieved by coordinating the activity of numerous different actin regulators to form the right structure at the right place and time within the cell. Through painstaking *in vitro* studies, we now know a lot about how purified actin regulators function in isolation. For example, the Arp2/3 complex generates dendritic networks of actin as opposed to the formins or Ena/VASP, which form linear, unbranched actin filaments 1, 2, 3. We also have an appreciation of how these actin assembly factors and the varying actin networks they generate contribute to the formation of different cellular structures. For instance, the branched actin meshworks arising from the Arp2/3 complex underlies lamellipod extension [4]. By contrast, filopods are formed from parallel bundles of actin filaments with formins or Ena/VASP molecules at their tips 5, 6, 7. We even have an idea how these structures support certain cellular processes, for example, the role of the lamellipod in driving a cell forward during migration. Thus, we have a growing understanding of how actin regulators give rise to specific structures and how these in turn allow cells to perform certain functions.

At the other end of the scale, we know that the actin cytoskeleton plays a key role in many aspects of human health and disease including embryonic development, cancer metastasis, wound repair, and inflammation. Each of these complex processes involves the coordinated formation of multiple actin-based structures. For instance, immune cell recruitment to sites of bacterial infection requires actin-driven chemotaxis, the extension of exploratory filopods to capture the pathogen, and the formation of phagocytic cups during engulfment 8, 9, 10. To achieve this, immune cells must be able to correctly deploy different combinations of actin regulators at the right time and place within the cell. Immune cells, in particular, require a remarkable amount of cytoskeletal plasticity to respond to a wide range of different stimuli 11, 12. As impressive as our progress has been, we still remain a long way from understanding how individual actin assembly factors and the structures they yield are deployed to promote complex cell behaviour. To make any headway in addressing this central question, it is important we move away from studying different actin regulatory pathways in isolation from one another and start exploring how they work as a collective. In other words we have to ask the question: how do the different actin assembly factors communicate and coordinate their efforts?

A number of recent publications have established the existence of a competition between different actin assembly factors for monomeric actin. By commanding a greater share of a finite pool of G-actin, actin regulators are able to limit each other's activities and therefore dictate what kind of actin networks and structures are formed. The details of these publications have been well reviewed elsewhere and therefore they will be only be summarised briefly here [13]. Instead, this review explores the possibility that competition is a general mechanism at work within the actin cytoskeleton. More specifically, we focus on whether or not cells are able to influence this competition and thus direct where and when one actin regulatory pathway dominates over the others. In such a scenario, subtle shifts in the balance of actin assembly factor activity would be sufficient to provoke wholesale rearrangements in the cytoskeleton. This in turn would confer the dynamism and plasticity necessary to drive complex cell behaviour similar to that observed in the cells of our immune system.

## Appetite for Competition: An Emerging Theme for Cytoskeletal Regulation

Ever since its initial discovery and characterisation, it has been accepted that the Arp2/3 complex is the driving force underlying lamellipod extension and cell motility 1, 14. Thus, when conclusive Arp2/3 complex deficient cells were eventually isolated, it was not entirely surprising that these cells lacked lamellipods 15, 16. What was not so easy to explain was why these cells instead extended excessive filopods. By their presence, these filopods confirmed that the Arp2/3 complex was not essential for these protrusions as had long been debated 7, 17. However it is not immediately obvious why the number of filopods should increase in the absence of the Arp2/3 complex.

**Figure 1:**
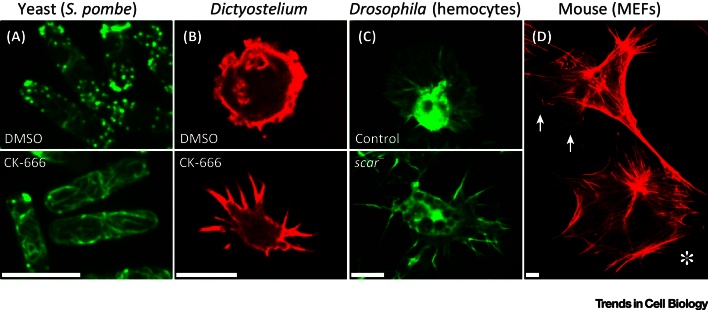
Increased Actin Bundle Formation following Disruption of the Arp2/3 Complex is an Evolutionary Conserved Cellular Response. (A) *Schizosaccharomyces pombe* expressing LifeAct-GFP and treated with either dimethyl sulfoxide (DMSO) or the Arp2/3 complex inhibitor CK-666. CK-666 treatment causes loss of Arp2/3 complex-dependent endocytic patches and an increase in actin cable formation (images obtained with permission from T.A. Burke and D.R. Kovar). (B) *Dictyostelium discoideum* expressing LifeAct-mRFP and treated with either DMSO or CK-666. Actin-rich ruffles are lost in *Dictyostelium* treated with CK-666 with filopods extended in their place (images obtained with permission from A.J. Davidson and R.H. Insall). (C) Loss of *scar* in LifeAct-GFP expressing *Drosophila* embryonic hemocytes results in lamellipod collapse and excessive filopod extension (images obtained with permission from A.J. Davidson and W. Wood). (D) Control and *arpC2* null (*) mouse embryonic fibroblasts (MEFs) fixed and stained with phalloidin. Disruption of the Arp2/3 complex causes lamellipod loss (arrows highlight lamellipods in control cell) and increased actin bundles in the form of filopods and stress fibres (images obtained with permission from J.D. Rotty and J.E. Bear). All scale bars represent 10 μm.

Disruption of SCAR/WAVE, the activator of the Arp2/3 complex at the leading edge, also promotes filopod formation at the expense of a lamellipod [17]. Furthermore, this phenotype is not confined to mammalian cell lines and is observed in SCAR-deficient *Drosophila* cells 18, 19. Similarly, Arp2/3 complex inhibition in *Dictyostelium discoideum* induces excessive filopod extension (Figure 1B). Together, these data imply that filopod formation in response to diminished Arp2/3 complex activity is an evolutionary conserved, intrinsic property of the actin cytoskeleton (Figure 1).

Such conservation lends itself to the adoption of simple, genetically tractable models and therefore it is not surprising that the breakthrough came from studies in fission yeast. *Schizosaccharomyces pombe* are immobile and thus have no need for any of the protrusions described earlier. Their cytoskeleton is essentially derived from just three different actin structures. These include short bursts of dendritic actin polymerisation occurring at sites of endocytosis, cables of actin that run through the cell, and the contractile actomyosin ring required for cytokinesis [20]. Whereas formins are required for the assembly of actin cables and the contractile ring, actin patches depend on the Arp2/3 complex 21, 22, 23, 24. The relatively simple actin cytoskeleton of *S. pombe*, therefore, makes for an ideal model to investigate how the Arp2/3 complex and formins influence one another and consequently the formation of these three, well-defined structures.

Recently, it was confirmed that inhibition of the Arp2/3 complex increased the activity of formins in *S. pombe* [24]. They demonstrated that the converse was also true, whereby the loss of both yeast formins increased actin patch density. Disruption of actin patch disassembly through depletion of ADF/cofilin impaired actin cable and contractile ring formation and suggested actin monomers were limiting. Together, this suggested that the Arp2/3 complex and formins compete with each other for G-actin and disruption of one frees more actin monomers for the other. Conclusively, the reduced or overexpression of actin itself was sufficient to stimulate the formation of one actin structure over the others. Low expression of actin favoured formin activity, whereas increased actin expression enhanced actin patch formation. Furthermore, the latter occurred at the expense of contractile ring assembly and resulted in impaired cytokinesis. The authors concluded that endogenous actin levels are such that the competing activities of the Arp2/3 complex and the formins are carefully balanced within cells.

In a follow-up study, the G-actin binding protein, profilin, was established as the pivot point within this homeostatic system [25]. Ectopically altering the ratio of actin to profilin in *S. pombe* revealed that profilin preferentially shunts G-actin to the formins and inhibits Arp2/3 complex-mediated actin polymerisation. It was further confirmed that profilin plays the same role in mammalian cell lines [26]. Microinjection of profilin 1 into murine fibroblasts caused lamellipod collapse and yielded cells that appeared like Arp2/3 complex-deficient cells in morphology. Conversely, profilin 1 knock-down increased lamellipod area. Finally, it was confirmed that excessive filopods of Arp2/3 complex-deficient cells are dependent on profilin 1 [26]. Although increasing profilin 1 levels in cells lacking the Arp2/3 complex had no effect, depletion of profilin 1 in these same cells reduced filopod length and number. Although these filopods were formed by Ena/VASP proteins rather than by formins, it was concluded that the loss of the Arp2/3 complex increased the pool of available G-actin for profilin 1 to be directed towards other actin regulators. The proline-rich motifs that profilin binds are far from exclusive to any one actin regulator and are present in formins, ENA/VASP proteins, and Arp2/3 complex-activating WASP family members. This does raise important questions as to how profilin is able to selectively divert actin monomers to one actin regulator over another. Instead, it was found that profilin actively inhibits Arp2/3 complex-mediated polymerisation while simultaneously favouring formin activity 25, 26. Profilin mutants unable to bind their proline-rich target sequences retained the ability to suppress the Arp2/3 complex, implying that profilin is more involved in cytoskeletal regulation than merely selectively bringing G-actin to one actin assembly factor as opposed to another. However, exactly how profilin inhibits the Arp2/3 complex requires further investigation.

**Figure 2:**
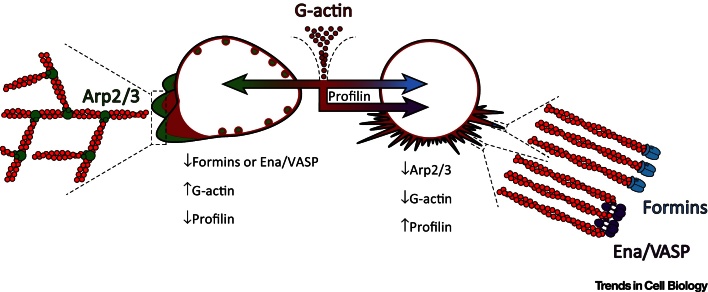
Actin Assembly Factors Compete with Each Other over a Finite Pool of G-Actin. Monomeric G-actin is either incorporated into dendritic networks of F-actin by the Arp2/3 complex or linear, unbranched networks by the formins and/or Ena/VASP. These actin assembly factors compete with one another over a limited supply of monomeric actin. The G-actin binding protein profilin maintains homeostasis within the actin cytoskeleton by ensuring the formins and/or Ena have access to this finite pool of actin monomers. This competition can be skewed experimentally by altering the levels of G-actin, profilin, or the actin assembly factors themselves. For instance, disruption of the formins in *Schizosaccharomyces pombe* or Ena/VASP in mammalian cell lines stimulates the Arp2/3 complex, resulting in increased dendritic actin networks and associated structures. Increased G-actin levels or suppressed profilin also has the same effect. Conversely, disruption of the Arp2/3 complex, reduced G-actin levels, or increased profilin levels stimulates the formins or Ena/VASP, promoting excessive actin cable or filopod formation depending on the organism studied.

As summarised in Figure 2, these papers demonstrate that the activities of the different actin assembly factors are kept in check through competition between each other for actin monomers. The inhibition of one of these actin regulators frees G-actin, fuelling the enhanced activity of the others. Homeostasis (and thus a dynamic, functional cytoskeleton) is maintained by profilin, which ensures that the pool of G-actin is not monopolised by the Arp2/3 complex through ring fencing monomers for other assembly factors such as formins. Profilin is unlikely to operate alone in this role and other actin regulators likely help maintain the equilibrium within the cytoskeleton. Thymosin β4, an alternative actin monomer binding protein, also appears to preferentially deliver cytosolic G-actin to formins rather than to the Arp2/3 complex within lamellipods of neuronally derived cell lines [27]. Another obvious candidate is capping protein, which caps growing actin filaments, preventing further elongation and thus preserving the pool of G-actin [28]. The branched actin networks generated by the Arp2/3 complex are highly sensitive to capping protein, whereas formins and Ena/VASP protect the filaments they form from capping 29, 30, 31, 32. Based on these *in vitro* data, it is possible that capping protein also diverts G-actin away from the Arp2/3 complex and towards other actin assembly factors. It must be noted, however, that capping protein has a far more complex role *in vivo* and has been found to be essential for lamellipod formation where it seemingly increases the activity of the Arp2/3 complex 33, 34.

Importantly, it is unlikely that competition is unique to the regulation of the Arp2/3 complex and its relationship with other actin regulators. It was found that the two *S. pombe* formins competed with each other for the increased G-actin made available following Arp2/3 complex inhibition [24]. The idea that competition is a general principle underlying the regulation of the actin cytoskeleton is consistent with several recent studies. For example, *Drosophila* Ena/VASP and Diaphanous-related formin (Ena and Dia, respectively) both localise to the tips of filopods extended by numerous, different motile cells within the fly 35, 36, 37. Although similar in appearance, filopods formed by Ena or Dia do have distinct dynamics consistent with their differing effects on polymerisation *in vitro* 38, 39. However, Dia and Ena seldom colocalise at the same filopod tip and, in the rare instances that they do, Ena appears to displace Dia and induce filopod retraction [39]. One interpretation of these data is that Dia and Ena are competing with one another for control of filopod tips; however, this remains to be fully explored.

## Embracing the Competition: A Means by Which to Dynamically Control Cell Behaviour

As described so far, competition between different actin regulators merely acts to support homeostasis within the cytoskeleton. Without profilin to counterbalance the activity of the Arp2/3 complex, formins and Ena/VASP would be deprived of actin monomers and structures such as contractile rings would fail to form. However, what if cytoskeletal competition offered more than simply maintaining a permissive state for certain actin regulators to function? What if this contest could be influenced within the cell to promote the formation of specific structures? Of course a single, all-encompassing competition between two actin regulatory pathways across the whole cell can only have a binary outcome and is at odds with the dynamic mix of actin-based structures present within cells. However, if this competition was instead played out independently between every individual actin assembly factor within the cell, the possible outcomes are infinite. What if cells were able to exert more subtle control over this contest, such as the local dampening of Arp2/3 complex activity so as to promote the extension of a single filopod? Rather than suppressing one actin regulatory pathway and independently activating another, what if cells were able to seamlessly induce one by inhibiting the other and vice versa?

**Figure 3:**
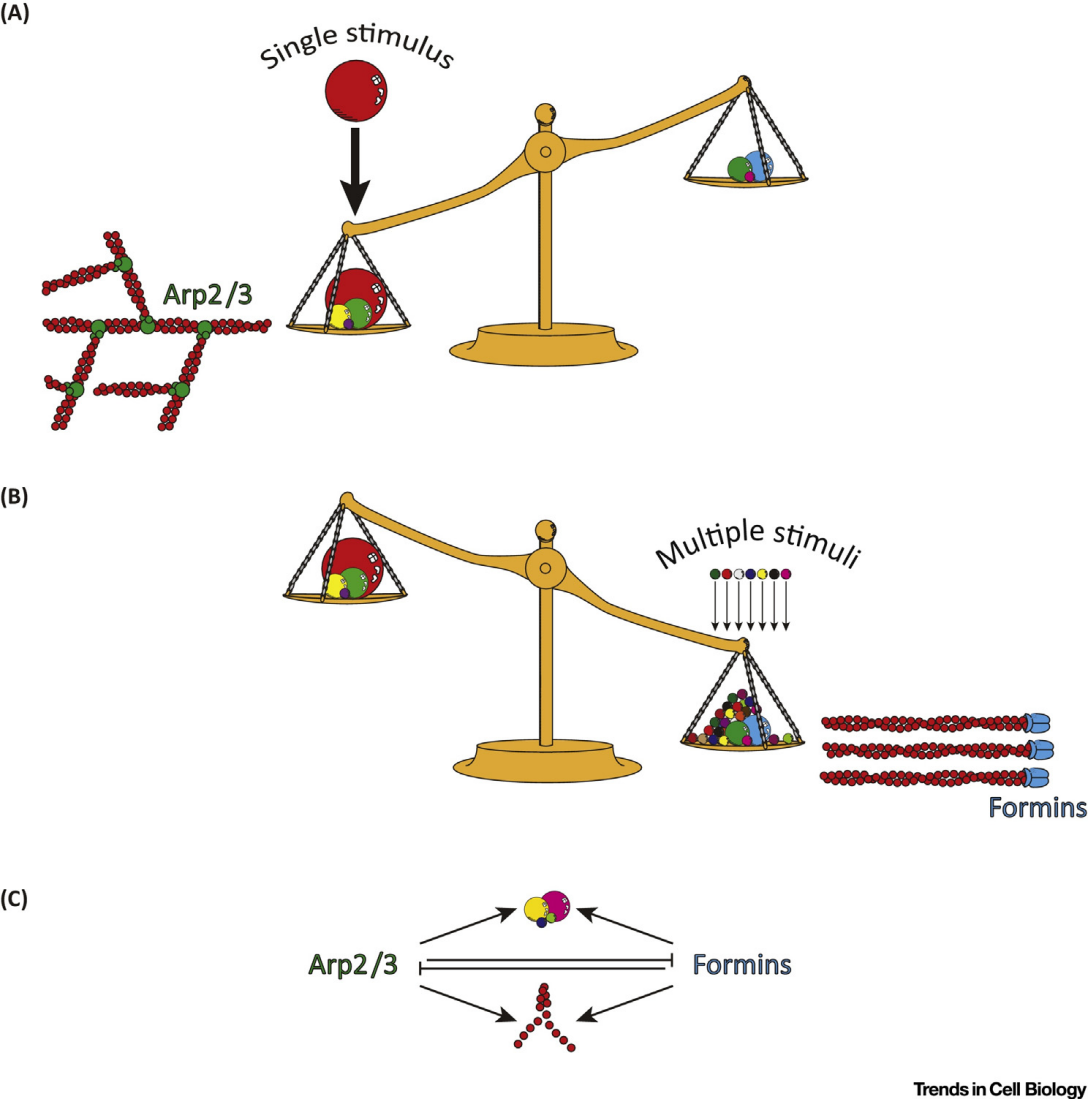
Key Figure: Cytoskeletal Competition as a Means to Balance and Integrate Multiple, Competing Stimuli

A cytoskeleton governed by a dynamic competition would be highly plastic and reactive, allowing cells to integrate and prioritise multiple conflicting signals. If imagined as a set of balance scales (Figure 3, Key Figure), the switch from one actin regulatory pathway to another would occur around a pivot point. The addition of weights (representing stimulatory signals) to one side would tip the scales in favour of one actin assembly factor over the other. Such a balance would be highly robust, capable of accommodating either single large inputs or integrating many smaller signals. Such scenarios are easily found in cell biology: during chemotaxis, leukocytes have to interpret shallow gradients of attractants via the heightened activation of receptors at one side of the cell compared with the other [40]. Conversely, processes such as T cell receptor engagement induce massive, unilateral actin polymerisation [41].

How could the competition between actin assembly factors be manipulated spatially and temporally within the cell to achieve such control? As previously suggested 13, 25, it is possible that profilin is selective in its ability to divert the flow of G-actin away from the Arp2/3 complex and towards formins and Ena/VASP. Exactly how profilin could achieve this is less certain. Different profilin isoforms or post-translational modifications could confer profilin some differential control 42, 43. Cells may also be able to vary the amount of profilin available to interact with G-actin through sequestration at the plasma membrane by phosphoinositides 44, 45. However, it is equally plausible that profilin and other actin regulators such as capping protein are constants that keep the actin cytoskeleton in equilibrium. In our balance scales analogy, they would act to set the pivot point upon which the balance beam rests. Artificially altering the ratio of profilin to actin would shift the fulcrum closer to one of the weighing pans, causing it to tip without the addition of any weight. In the normal setting, however, the profilin to actin ratio might vary by little, offering minor opportunity for profilin to direct cytoskeletal rearrangements.

Other than G-actin, actin regulators could be competing for upstream activators or mediated by direct interaction between assembly factors themselves. Starting with the former, certain actin regulators are known to interact with the same signalling molecules. For example, the Rho GTPase Rac activates the Arp2/3 complex in lamellipod via the SCAR complex 46, 47. However, both *Dictyostelium* and mammalian Diaphanous-related formins have also been shown to interact with Rac 5, 48, 49. If active and GTP-bound Rac was limiting, competition between these two actin assembly factors would be expected. Differential activation could then be achieved through different coactivators working with Rac to skew the competition in favour of the Arp2/3 complex over the Diaphanous-related formins or vice versa.

Alternatively or in addition to this, actin regulators could influence each other's activity through direct interactions. The SCAR complex, Diaphanous-related formins, and Ena/VASP interact with one another 39, 50, 51, 52, 53, 54, and two of these interactions have an inhibitory effect. For instance, Ena appears to directly bind and suppress the activity of Dia at the tips of filopods in *Drosophila* cells [39]. If these proteins do cross-inhibit each other through such interactions, a dynamic competition would exist within the actin cytoskeleton. Regardless of the number or type of stimuli, if two actin regulatory pathways were activated to the same extent they would cancel out each other's activity. However, if the activity of one were even slightly dampened, the other would be released from constraint. The newly dominant actin assembly factor would strongly suppress the other actin regulators and monopolise the pool of available G-actin. If this competition were conducted at the subcellular scale, it could drive the formation of new actin-based structures in specific regions of the cell, which in turn could promote a change in cell behaviour. Importantly, this form of regulation would occur at the level of the actin regulators themselves. A disparate number of stimulatory signals would be funnelled down to the individual actin assembly factors. Signal integration and prioritisation of competing cues would be achieved through the competition between these actin regulators, with the resulting decision being enacted by the winner. Ultimately, this would allow actin assembly factors to coordinate their activity to promote changes in cell shape and behaviour.

## Keeping Up with the Competition: Concluding Remarks

As a means of controlling and coordinating the cytoskeleton, competition offers many advantages. An actin cytoskeleton derived from a dynamic contest between actin regulators would be highly responsive to stimuli, be they small or large, consonant or conflicting. Although the initial studies reviewed here have focused on the role of competition in the nucleation and elongation of F-actin, it will be interesting to explore whether it also contributes to other aspects of cytoskeletal regulation such as actin network remodelling and turnover. However, the dynamism inherent to such an arrangement leaves it incredibly difficult to study. The long-term inactivation of individual actin assembly factors is a crude tool to dissect the mechanisms mediating competition because the unconditional loss of one actin regulator severely skews the competition in favour of another assembly factor. This unconstrained activity inevitably overwhelms the cytoskeleton and locks it in an exaggerated and paralysed configuration. For example, the excessive filopods extended when the Arp2/3 complex is inhibited. It is the moments immediately after equilibrium is disturbed that will reveal the most about how this competition actually works. This will require new technologies to allow us to spatially and temporally manipulate the actin cytoskeleton. Of equal importance is the setting in which this competition is to be explored. The apparent universality of this competition and the complexity of the actin cytoskeleton favour the adoption of simple, genetically tractable models. Furthermore, given their crucial role in establishing cytoskeletal competition in the first place, model organisms will surely prove indispensable.

As highlighted in the Outstanding Questions, we do not yet know the true extent to which competition underlies the actin cytoskeleton. However, with further exploration it could hold the key to unifying what we know about the biochemistry of individual actin regulators with how they are deployed as a collective to drive cellular behaviour.

Outstanding Questions How general a theme is competition within the actin cytoskeleton? If so, how many other actin assembly factors are regulated by such means?

Does competition play a role beyond actin nucleation and elongation? Is it involved in all aspects of cytoskeletal regulation such as actin turnover?

Other than profilin, what other actin regulators help mediate cytoskeletal competition?

Do actin assembly factors compete solely for G-actin or are there other limiting factors, such as activators? Or do they influence one another's activity through direct interactions?

Does cytoskeletal competition operate at the subcellular, yielding different outcomes in different regions within the cell? If so, can cells spatially and temporally influence competition to promote changes in cell behaviour?
